# Pictorial Review of Lesion Localization for Patients With Stroke, Upper Limb and Lower Limb Pathology

**DOI:** 10.1212/NE9.0000000000200012

**Published:** 2022-10-14

**Authors:** Sarah Lyon

**Affiliations:** From TCU School of Medicine, Fort Worth, Texas.

Localization is a foundational skill in clinical neuroscience and a vital aspect of teaching and learning for students and educators. Stroke syndromes, brachial plexus injury, and lumbosacral nerve pathology are among some of the most challenging clinical scenarios for learners to understand and master. The 3 images in this article are teaching tools for learners and teachers in clinical neuroscience. The “Vascular Localization of Stroke” image is a reference tool for learning cerebrovascular anatomy and the effect of a cerebrovascular accident, according to the location of the lesion. The “Upper Limb Pathology” and “Lower Limb Pathology” images are handout tools for learning how lesions to peripheral nerves or spinal nerve roots present with sensory and/or motor deficits.

## Vascular Localization of Stroke


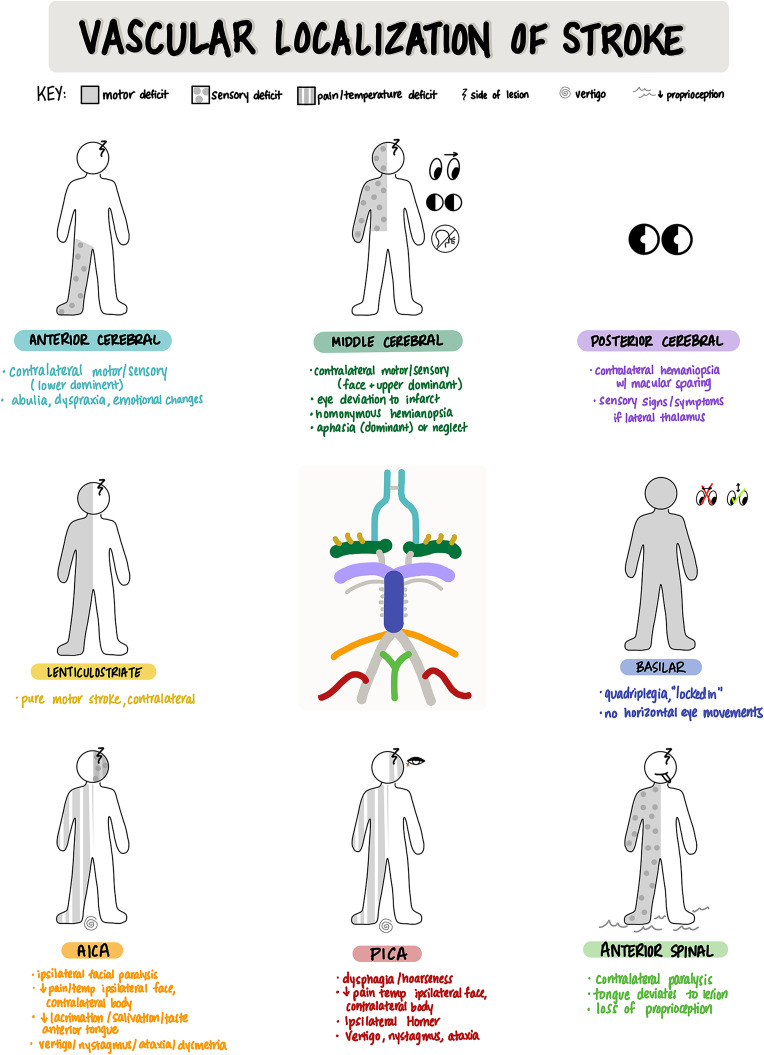


## Upper Limb Nerve Pathology


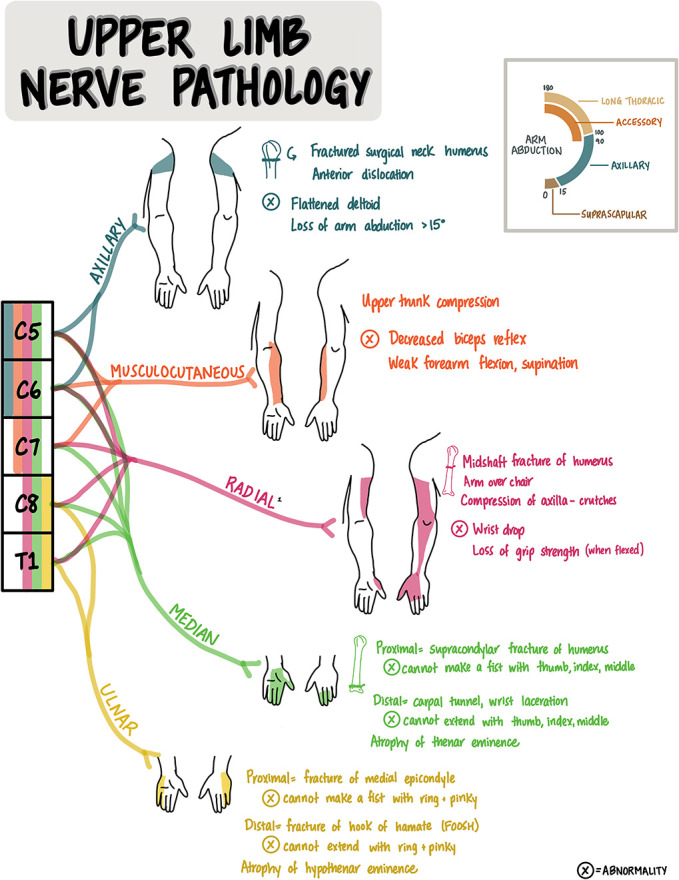


1.Glover
NM, Murphy
PB. Anatomy, shoulder and upper limb, radial nerve [updated 2021 Jul 27]. In: StatPearls [Internet]. StatPearls Publishing, 2022. ncbi.nlm.nih.gov/books/NBK534840/.30521261

## Lower Limb Nerve Pathology


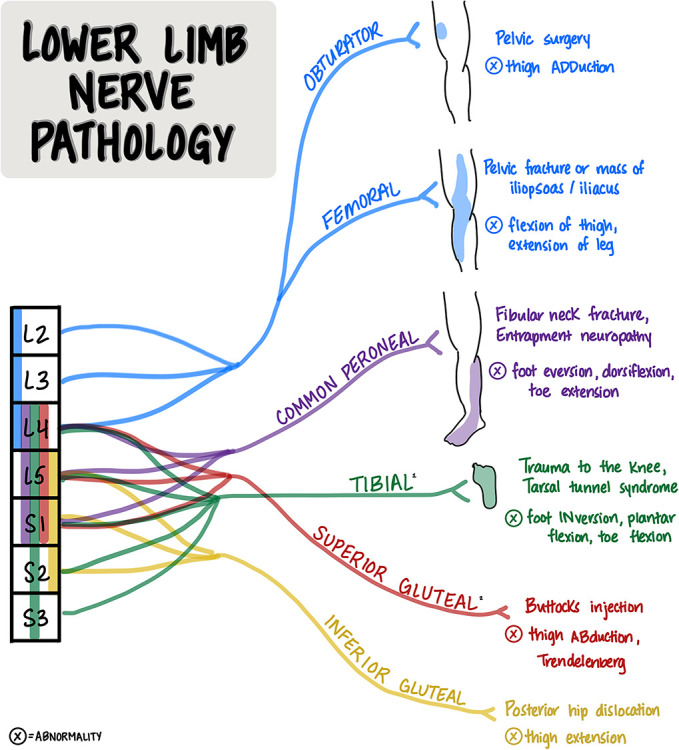


1.Desai
SS, Cohen-Levy
WB. Anatomy, bony pelvis and lower limb, tibial nerve [updated 2021 Aug 11]. In: StatPearls [Internet]. StatPearls Publishing, 2022. ncbi.nlm.nih.gov/books/NBK537028/.307257132.Lung
K, Lui
F. Anatomy, abdomen and pelvis, superior gluteal nerve [updated 2021 Aug 11]. In: StatPearls [Internet]. StatPearls Publishing, 2022. ncbi.nlm.nih.gov/books/NBK535408/.30571029
